# Evaluation of Probiotic and Antimicrobial Properties of Patulin-Degrading *Latilactobacillus sakei* KMP17 and Its Fermentation

**DOI:** 10.3390/foods15020234

**Published:** 2026-01-09

**Authors:** Zi-Qi Yang, Xin-Ru Wen, Chun-Zhi Jin, Taihua Li, Feng-Jie Jin, Hyung-Gwan Lee, Long Jin

**Affiliations:** 1 Co-Innovation Center for Sustainable Forestry in Southern China, College of Ecology and Environment, Nanjing Forestry University, Nanjing 210037, China; 2 Cell Factory Research Centre, Korea Research Institute of Bioscience & Biotechnology (KRIBB), Daejeon 34141, Republic of Korea

**Keywords:** fermentation optimization, lactic acid bacteria, patulin biodegradation, probiotic properties

## Abstract

Lactic acid bacteria (LAB), as significant probiotics, hold immense application potential across diverse fields. This study systematically evaluated the probiotic properties and patulin degradation capabilities of four LAB strains with potent antimicrobial effects, previously isolated from Kimchi: *Weissella cibaria* (KM4 and KM14), *Latilactobacillus sakei* KMP17, and *Leuconostoc mesenteroides* KM35. All exhibited favorable environmental tolerance, adhesion capacity, and safety, along with the potential to degrade patulin. Out of these, *L. sakei* KMP17 demonstrated outstanding probiotic characteristics, high safety, and PAT degradation potential. Further investigation revealed that viable cell metabolism is the primary mechanism for PAT degradation by *L. sakei* KMP17, and PAT induction was hypothesized to stimulate the production of specific degradation enzymes. Concurrent whole-genome sequencing confirmed the high safety and significant probiotic potential of *L. sakei* KMP17. This research provides high-quality candidate strains and a theoretical foundation for the application of LAB in the field of food mycotoxin biodegradation.

## 1. Introduction

Patulin (PAT), a highly toxic fungal secondary metabolite, is commonly found in cereals, fruits, and fruit-derived products (e.g., juices, jams, and preserves), and is closely associated with fruit “brown rot” or other decay characteristics. In recent years, incidents of PAT contamination exceeding regulatory limits in food or feed have been frequently reported both domestically and internationally. For instance: In 1962, Japanese dairy cows accidentally consumed PAT-contaminated feed, leading to a large-scale mortality event [1]. And in 2010, a European study reported that 14 out of 35 pear juice samples exceeded the maximum allowable limit of 50 μg/kg [2]. What is more, in 2017, the Jiangxi Provincial Food and Drug Administration tested 36 batches of fruit products, identifying 2 non-compliant batches due to excessive PAT levels. These recurring incidents highlight the persistent risks posed by PAT contamination, underscoring the urgent need for enhanced monitoring and regulatory measures to ensure food safety.

*Penicillium expansum*, the primary producer of PAT, predominantly infects fruits such as apples, oranges, peaches, bananas, strawberries, and cherries. Following infection by *P. expansum*, fruits undergo decay accompanied by PAT production. After accumulating in decayed tissues, 2–6% of PAT diffuses into adjacent healthy tissues. Notably, PAT exhibits high solubility in water, acid resistance, and thermal stability, rendering it highly persistent and difficult to eliminate during food production, transportation, and storage, thereby posing significant contamination risks [3]. Furthermore, toxigenic strains of *P. expansum* readily colonize cereals and vegetables, making PAT contamination a global challenge for the food industry. Worldwide surveys have documented widespread PAT contamination: In Italy, 34.8% of tested apple and mixed fruit juices were found to be contaminated with PAT [4]. A Spanish study detected PAT in 23 out of 58 concentrated juice samples, with the highest levels observed in pear and apple concentrates, though all remained below EU regulatory limits [5]. Poostforoushfard et al. [6] identified PAT in 55 out of 75 apple juice and canned fruit samples collected in Iran. In China, a survey of 135 dried fruits, juices, and jams revealed a PAT contamination rate of 30.7%, with 24 samples exceeding the 50 μg/kg threshold [7]. These findings underscore the pervasive nature of PAT contamination across diverse geographical regions and food matrices.

Mycotoxin contamination in cereal crops inflicts substantial economic losses on agricultural systems. Globally, approximately 25% of grain production is contaminated by mycotoxins annually [8]. As a major agricultural producer, China ranks among the countries most severely affected by mycotoxin contamination. According to incomplete statistics, annual grain losses in China due to mycotoxin contamination account for approximately 6% of total production [9]. Given the dual threats of PAT to human health and economic stability, mitigating PAT contamination and developing effective control strategies have garnered increasing attention over recent decades. Researchers have persistently explored solutions, with the safe and efficient removal of PAT from food and feed remaining a focal research area. Current methods for controlling PAT contamination are categorized by their mechanisms into physical, chemical, and biological approaches.

Porous materials, such as gelatin, resins, calcium alginate, and activated carbon, exhibit adsorption capabilities toward PAT and are widely applied in liquid food systems [10]. For instance, Diao et al. [11] demonstrated that ultraviolet irradiation (254 nm) of apple juice achieved a PAT degradation rate of 82.92%. Sulfur dioxide (SO_2_), a common fruit preservative, has also been shown to reduce PAT levels in apple juice by 33–100% at 30 °C (250 μg/mL SO_2_). Notably, when stored at 4 °C, PAT concentrations in juice can decline below detectable limits [12]. While physical and chemical methods for PAT removal are extensively studied, they often suffer from limitations such as prolonged processing times, high costs, and adverse effects on product quality. In contrast, biological approaches, which utilize microorganisms or enzymes to reduce PAT levels, are categorized into biosorption and biodegradation based on their mechanisms. These methods offer advantages including high specificity, non-toxic byproducts, and mild operating conditions.

Yeasts are prominent agents in fungal-mediated PAT degradation: Burroughs et al. [13] reported that yeast fermentation removed 90% of PAT. Shao et al. [14]. identified six PAT degradation products generated during yeast fermentation using GC-MS analysis. Antagonistic microorganisms, emerging as a novel strategy for mycotoxin control, have gained attention due to their efficacy. Certain strains effectively degrade PAT under in vitro conditions: Wei et al. [15] screened multiple LABs and found *Bifidobacterium animalis* to exhibit the highest PAT degradation activity (80% reduction in liquid media). A *Lactiplantibacillus pentosus* strain reduced PAT levels in apple juice by 53.14% within 24 h, with stability tests confirming degradation as the primary mechanism [16]. Luo et al. [17] isolated *Pichia pastoris* S15-8, which degraded over 90% of PAT in apple juice and demonstrated tolerance to high PAT concentrations (up to 100 mg/L in culture media).

Lactic acid bacteria (LAB) reduce PAT primarily through cell wall adsorption and intracellular/extracellular enzymatic degradation [18]. Bahati et al. [19] investigated PAT adsorption by LAB and revealed that alkaline amino acids, thiols, and ester compounds—rather than the peptidoglycan layer—are critical determinants of PAT adsorption, primarily involving interactions between PAT and functional groups such as C=O, N−H, C−H, and N−O. Additionally, PAT removal from apple juice by *Lactobacillus plantarum* and *Lactobacillus acidophilus* correlates with the presence of probiotics [20]. For instance, supplementation with fructo-oligosaccharides and ascorbic acid during 6-week cold storage enhanced LAB-mediated PAT elimination, achieving a maximum clearance rate of 91.23%.

Microbial or enzymatic degradation of PAT involves disruption of its lactone ring or hemiacetal ring, yielding less toxic metabolites such as desoxypatulinic acid and E/Z-ascladiol [21]. LAB further degrade PAT via heat-labile extracellular or intracellular factors that cleave its pyran ring, producing E-ascladiol and Z-ascladiol [22]. *Lactobacillus casei* YZU01, when induced by PAT, secretes extracellular enzymes capable of efficiently degrading PAT, playing a pivotal role in toxin elimination [23].

This study aims to evaluate the safety and PAT-degrading potential of LAB strains previously screened for their antagonistic activity against *P. expansum*, thereby identifying candidate strains for PAT biodegradation; investigate the mechanisms underlying PAT degradation by LAB to elucidate the biochemical pathways involved; obtain complete genomic data through whole-genome sequencing technology, enabling molecular-level analysis of functional genes (e.g., probiotic traits, antibiotic resistance genes) and providing genetic insights into strain functionality; and assess practical applicability by correlating genomic features with observed PAT degradation efficiency and safety profiles, thereby supporting the development of LAB-based biocontrol strategies in food safety.

## 2. Material and Methods

### 2.1. Identification of Antifungal Lactic Acid Bacteria (LAB) and Evaluation of Their Application Potential

#### 2.1.1. Source, Isolation, and Identification of Antifungal LAB

The four LAB strains used in this study were previously isolated from Kimchi in our laboratory. Genomic DNA was extracted from the strains using the cetyltrimethylammonium bromide method. The 16S rRNA gene fragments of the strains were amplified via polymerase chain reaction (PCR) with the following primers: Forward primer 27F: 5′-AGAGTTTGATCCTGGCTCAG-3′; Reverse primer 1492R: 5′-GGTTACCTTGTTACGACTT-3′. The amplified sequences were then analyzed to construct a phylogenetic tree using the neighbor-joining method [24].

#### 2.1.2. Acid-Alkali Tolerance and Surface Properties

Acid-alkali tolerance and surface hydrophobicity of the four strains were systematically evaluated. For acid tolerance testing, the pH of MRS broth was adjusted to 2.0 and 4.0 using 1 mol/L HCl and 1 mol/L NaOH, respectively. Alkali tolerance was assessed using MRS broth supplemented with 0.1% and 0.3% oxgall (bile salts). Surface hydrophobicity was determined by mixing treated bacterial suspensions with chloroform or xylene at a 1:3 ratio (1 mL bacterial suspension: 3 mL organic solvent), followed by static incubation at 37 °C for 3 h. The optical density (OD_600_) of the aqueous phase was measured, and hydrophobicity was calculated using Formula (1). For auto-aggregation analysis, treated suspensions were sampled at 3 h, 12 h, 24 h, 36 h, and 48 h, with OD_600_ measurements of the aqueous phase used to calculate auto-aggregation capacity via Formula (2). All experiments were conducted in triplicate (three biological replicates).

(1) $$Hydrophobicity\;(\%)=\frac{A_{i}-A_{f}}{A_{i}}\times 100\%$$

(2) $$Auto-Aggregation\;(\%)=\frac{A_{0}-A_{t}}{A_{0}}\times 100\%$$

where $A_{i}$ : the absorbance at OD_600_ of the initial bacterial suspension; $A_{f}$ : the absorbance at OD_600_ after mixing with the organic solvent and standing at 37 °C for 3 h; $A_{0}$ : the absorbance at OD_600_ of the initial bacterial suspension at 0 h; $A_{t}$ : the absorbance at OD_600_ of the bacterial suspension measured at five different time points.

#### 2.1.3. Safety Assessment and Antagonistic Activity of LAB Strains

The safety profiles of the four strains were evaluated through antibiotic susceptibility testing and hemolytic activity assays. For antibiotic susceptibility, antibiotic disks (various types) were placed on MRS agar plates inoculated with the strains, followed by incubation at 37 °C for 24 h. The diameters of inhibition zones were measured to determine resistance profiles. Hemolytic activity was assessed by streaking single colonies onto Columbia blood agar plates and incubating at 30 °C for 24 h. The absence of hemolytic zones indicated non-hemolytic activity, with *Staphylococcus aureus* ATCC-9144 (showing hemolysis) serving as a positive control.

Numerous studies have demonstrated that LAB fermentation can produce antimicrobial proteins or employ competitive exclusion mechanisms to inhibit the growth of pathogens [25,26]. To assess the inhibitory effects of the four isolated LAB strains on common pathogenic bacteria, an antimicrobial activity assay was conducted against *Staphylococcus aureus* and *Listeria monocytogenes*. Cell-free supernatants (CFS) were obtained by filtering cultures at different time points through 0.22 μm sterile membranes. The CFS was then tested against *Staphylococcus aureus*, *Staphylococcus aureus* FBS200, and *Listeria monocytogenes* using the agar well diffusion assay. All experiments were performed in triplicate. In the agar well diffusion assay, the central well was filled with 100 μL of MRS broth as a blank control, while the remaining four wells were supplemented with CFS collected at different cultivation periods. Specific labeling for individual wells was omitted for brevity.

### 2.2. Screening of Patulin-Degrading LAB and Degradation Analysis

#### 2.2.1. Quantification of PAT in Fermentation Broth

Four LAB strains were subcultured for 2–3 generations, adjusted to OD_600_ = 1.0, and inoculated (1% *v*/*v*) into MRS broth containing 40 μg/mLPAT. A control group (MRS broth with 40 μg/mL PAT, without LAB inoculation) was included. All samples were incubated statically at 30 °C for 24 h, followed by PAT extraction and quantification via high-performance liquid chromatography (HPLC).

For extraction, 1 mL of culture was centrifuged at 10,000 rpm for 5 min (4 °C), and the supernatant was extracted twice with 2 mL ethyl acetate. The combined organic phase was evaporated to dryness under nitrogen gas, reconstituted in 1 mL of 10% acetonitrile, and filtered through a 0.22 μm membrane prior to HPLC analysis. PAT quantification followed the method by Zhang et al. [27], utilizing a standard curve correlating PAT concentration to chromatographic peak area. The PAT content in samples was calculated using Formula (3).

(3) $$PAT(\mu g/mL)=\frac{C\times V}{M}\times 100\%\;$$

where: *C*: Concentration of PAT in the test solution (μg/mL); *V*: Volume of the sample extract (mL); *M*: Volume of the test solution (mL).

#### 2.2.2. PAT Removal Capacity Assay

The PAT content was quantified using a Waters Alliance e2695 HPLC system (Waters Corporation, Milford, MA, USA), following the methodology described in Section 2.2.1. The HPLC parameters and detection protocol remained unchanged. A standard curve correlating PAT concentration (μg/mL) to chromatographic peak area was established, enabling the determination of PAT content in test samples based on the linear regression model derived from the curve.

##### Determination of PAT Removal Capacity by Viable and Heat-Inactivated Cells

For the Viable Cells, the strains were subcultured for 2–3 generations, adjusted to OD_600_ = 1.0, and inoculated (1% *v*/*v*) into MRS broth containing 5 μg/mL PAT. A 0 h sample served as the control (Ck). Following static incubation at 37 °C for 24 h, the culture (L_0_) was filtered through a 0.22 μm membrane for HPLC analysis.

For the Heat-Inactivated Cells, bacterial cells were inactivated by heating at 100 °C for 30 min in a water bath. The inactivated cells were resuspended in PBS buffer containing 1 μg/mL PAT (L0), with a PAT-containing PBS buffer (no cells) as the control (Ck). After static incubation at 37 °C for 24 h, samples were centrifuged at 12,000 rpm for 3 min, and the supernatant was filtered (0.22 μm) for HPLC analysis.

PAT concentrations in both groups were determined via HPLC using a pre-established standard curve. All experiments were performed in triplicate (three biological replicates).

##### Determination of PAT Removal Capacity by Bacterial Components

For the Extracellular Components, LAB cultures were adjusted to OD_600_ = 1.0 and inoculated (1% *v*/*v*) into 10 mL MRS broth containing 10 μg/mL PAT (induced group) or without PAT (non-induced group). After static incubation at 30 °C for 16 h, cultures were centrifuged at 5000 rpm for 5 min to collect cells and supernatants. Supernatants from both groups were filtered (0.22 μm), supplemented with 1 μg/mL PAT, and analyzed via HPLC at 0 h (Ck control). Samples were then incubated statically at 37 °C for 24 h, centrifuged (12,000 rpm, 3 min), and filtered (0.22 μm) for PAT quantification.

For the Intracellular Components and Cell Walls, cells from non-induced and induced groups were washed twice with PBS, resuspended in 5 mL PBS to equalize OD_600_, and disrupted via sonication (300 W, 3 s on/2 s off cycles). Lysates were centrifuged at 6000 rpm for 10 min (4 °C) to separate intracellular components (supernatant) and cell wall residues (pellet resuspended in PBS). Both fractions were supplemented with 1 μg/mL PAT (L_0_), while PBS containing 1 μg/mL PAT served as the control (Ck). After 24 h incubation at 37 °C, samples were centrifuged (12,000 rpm, 3 min) and filtered (0.22 μm). PAT concentrations in all groups were quantified via HPLC using a pre-established standard curve. Triplicate biological replicates were performed for each assay.

### 2.3. Establishment of PAT Standard Curve by HPLC Method

PAT standard solutions with concentrations of 2 μg/mL, 5 μg/mL, 10 μg/mL, 20 μg/mL, and 50 μg/mL were prepared and analyzed using an Agilent 1260 high-performance liquid chromatography (HPLC) system. A standard curve was constructed by plotting PAT concentrations (*x*-axis) against the corresponding HPLC peak areas (*y*-axis), as shown in Supplementary Figure S1. The linear regression equation was determined as y = 11.07x + 1.282 with a correlation coefficient (R^2^) of 0.9999, indicating an excellent linear relationship between PAT concentration and peak area. This validated method demonstrates sufficient reliability for PAT quantification in subsequent experiments, and the established standard curve was applied for screening lactic acid bacteria with PAT-degrading capabilities.

### 2.4. Standardization of Fermentation Conditions for Strain KMP17

Fermentation parameters (inoculum size, temperature, medium volume, agitation speed, and pH) for KMP17 were standardization.

Cultures adjusted to OD_600_ = 1.0 were inoculated at 1%, 3%, and 5% (*v*/*v*) into MRS broth and incubated statically at 30 °C, with samples collected at 12 h, 24 h, and 48 h; temperature optimization involved static incubation at 25 °C, 30 °C, and 37 °C, sampled at identical intervals; Medium volume effects were assessed using 5 mL, 10 mL, and 14 mL MRS broth in 15 mL tubes, and 20 mL in 50 mL flasks; Agitation conditions included static incubation (30 °C) versus shaking at 100 rpm and 200 rpm (30 °C); pH optimization tested initial MRS broth pH values of 2, 4, 6, and 8. Post-sampling, OD_600_ was measured via a multifunctional microplate reader to evaluate viable cell density, while cell-free supernatants (CFS) were retained for antifungal assays against *P. expansum* was cultured on PDA agar at 25 °C for 5 d, and spores were eluted with 15 mL sterile buffer, homogenized, and diluted to 10^4^ CFU/mL using a hemocytometer.

Following Ruggirello et al. [28] with modifications, 100 μL spore suspension and 100 μL LAB-CFS were co-incubated in 96-well plates at 25 °C for 72 h. OD_490_ (ODt) was measured, with controls including 100 μL spore suspension add 100 μL MRS broth (COD) and 100 μL MRS broth add 100 μL sterile eluent (blank). Triplicate biological replicates were performed.

### 2.5. Whole-Genome Sequencing and Functional Annotation

The whole-genome sequencing of *L. sakei* KMP17 was conducted by Shanghai Majorbio Bio-pharm Technology Co., Ltd (Shanghai, China). Enriched cultures were centrifuged to harvest cells, and genomic DNA was extracted using an optimized SDS-based DNA extraction protocol. Following quality assessment, sequencing, assembly, and quality control were performed. The assembled genome was annotated by the National Center for Biotechnology Information (NCBI), including predictions of coding genes, non-coding RNAs, repetitive sequences, and CRISPR elements. Functional annotation of coding sequences was performed using multiple databases: the NR database for taxonomic classification, GO database for protein functional annotation, COG database for gene categorization, KEGG database for metabolic pathway prediction, CAZy database for carbohydrate-active enzyme (CAZyme) identification, and CARD for antibiotic resistance gene screening [29]. Secondary metabolism in microorganisms occurs during specific growth phases (typically the stationary phase), where primary metabolites are utilized as precursors to synthesize compounds with no direct role in immediate survival, termed secondary metabolites. These metabolites are generally regulated by multi-gene clusters encoding multifunctional enzymes. Among these, polyketide synthases (PKS) and nonribosomal peptide synthetases (NRPS) are the most extensively studied classes. The antiSMASH database (https://antismash.secondarymetabolites.org/; accessed on 9 June 2025) enables rapid identification, annotation, and analysis of secondary metabolite biosynthetic gene clusters (BGCs) in microbial genomes [30]. Secondary metabolite biosynthesis gene clusters were predicted via the antiSMASH database, with additional analyses using Pfam (protein families), VFDB (virulence factors), and ResFinder (antimicrobial resistance genes).

## 3. Results

### 3.1. Identification of Lactic Acid Bacteria

The strains KM4, KM14, KMP17, and KM35 were identified via 16S rRNA sequencing, with results aligned against the NCBI database using BLAST version 2.17.0 (Table S1). Strains KM4 and KM14 were confirmed their classification as *W. cibaria*. Strain KMP17 was identified it as *L. sakei*, with its phylogenetic tree reconstructed (Figure S2). Similarly, strain KM35 was confirmed as *Leuconostoc mesenteroides*.

### 3.2. Probiotic Evaluation of L. sakei KMP17

#### 3.2.1. Acid and Bile Salt Tolerance Under Simulated Gastrointestinal Conditions

**Figure 1:**
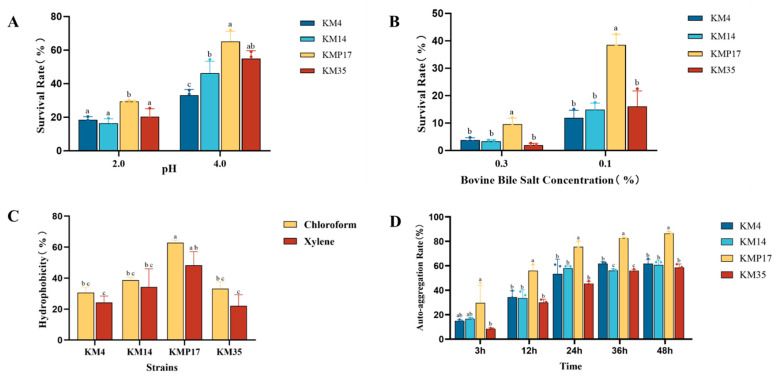
Tolerance and surface properties analysis of LAB under Simulated Gastrointestinal Conditions. Survival rate of the KMP17 under different pH conditions (**A**); survival rate of the *L. sakei* KMP17 at different bile salt concentrations (**B**); hydrophobicity of the *L. sakei* KMP17 (**C**); auto-aggregation rate of the *L. sakei* KMP17 (**D**). Different letters on the error bars indicate statistically significant differences within the group (*p* < 0.05).

As shown in Figure 1A, LAB strains exhibited acid tolerance, though with significant differences in survival rates (*p* < 0.05). While all strains survived in MRS broth at pH 2.0, their viability was relatively low. Strain *L. sakei* KMP17 demonstrated the strongest acid tolerance, with a survival rate of 29.46 ± 0.58%, significantly higher than the other three strains (*p* < 0.05). At pH 4.0, survival rates improved for all strains, with *L. sakei* KMP17 retaining the highest viability (65.23 ± 5.93%). The survival rates (>33% at pH 4.0) suggest their potential to withstand gastric acidity and colonize the intestinal tract.

For bile salt tolerance (Figure 1B), strains were assessed via plate counting in MRS broth containing 0.1% or 0.3% oxgall. In 0.1% oxgall, *L. sakei* KMP17 exhibited the highest survival rate (38.46 ± 3.92%, *p* < 0.05). At 0.3% oxgall, survival rates declined sharply: *L. sakei* KMP17 retained 9.60 ± 2.18% viability, whereas the other strains fell below 5%.

All strains displayed moderate tolerance to simulated gastrointestinal conditions, with *L. sakei* KMP17 demonstrating superior acid and bile salt resistance. These findings highlight *L. sakei* KMP17’s robust survival capability in gastric and intestinal environments, underscoring its potential as a probiotic candidate.

#### 3.2.2. Hydrophobicity and Auto-Aggregation Capacity

The surface hydrophobicity of the strains toward chloroform and xylene is shown in Figure 1C. *L. sakei* KMP17 displayed superior auto-aggregation rates in both chloroform and xylene, achieving surface hydrophobicity values of 62.8 ± 1.7% in chloroform and 48.28 ± 8.88% in xylene.

As illustrated in Figure 1D, the auto-aggregation capacity of the strain increased over time, stabilizing after 24 h. Notably, *L. sakei* KMP17 demonstrated a strong auto-aggregation performance, achieving values of 56.01 ± 4.8% at 12 h, 75.66 ± 4.43% at 24 h, and 86.51 ± 1.68% at 48 h.

### 3.3. Safety Analysis: Antibiotic Tolerance and Hemolysis

The antibiotic susceptibility of the four selected LAB strains to 14 common antibiotics is summarized in Table S2. The four LAB strains exhibited variability in their susceptibility profiles: all strains were sensitive to ampicillin/sulbactam, tetracycline, and erythromycin, resistant to nalidixic acid and vancomycin, and displayed intermediate resistance to lincomycin, chloramphenicol, and rifampicin.

The four strains were streaked onto Columbia blood agar plates, with *S. aureus* ATCC9144 as a positive control, to evaluate hemolytic zones (Figure S3). Strains KM4 and KM14 exhibited α-hemolysis. The positive control (*S. aureus* ATCC9144) and strain KM35 demonstrated *β*-hemolysis. *γ*-hemolysis (non-hemolytic), with no visible halo around colonies, was observed in strain *L. sakei* KMP17, confirming its non-hemolytic nature.

### 3.4. Antimicrobial Activity

**Figure 2:**
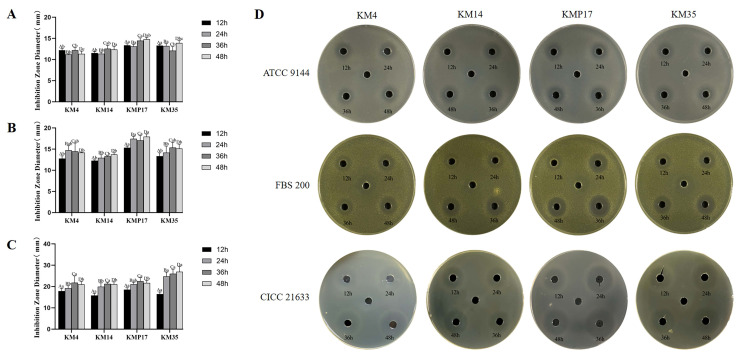
Evaluation of Antimicrobial Activity. Antagonistic activity of strain supernatants against *S. aureus* ATCC 9144 (**A**); Antagonistic activity of strain supernatants against *S. aureus* FBS 200 (**B**); Antagonistic activity of strain supernatants against *L. monocytogenes* CICC 21633 (**C**); The inhibitory effects of cell-free supernatants from four bacterial strains against three different pathogenic bacteria. The horizontal axis displays the four distinct strains: KM4, KM14, KMP17, and KM35, while the vertical axis represents the three pathogenic bacteria: *S. aureus* ATCC 9144, *S. aureus* FBS 200, and *L. monocytogenes* CICC 21633. The figure clearly shows the sizes of the inhibition zones produced by the cell-free supernatants against the pathogenic bacteria at four time points: 12 h, 24 h, 36 h, and 48 h (**D**). The labels A, B, C, and D indicate different groups, and different lowercase letters (a, b, c) denote statistically significant differences within the group level (*p* < 0.05).

The four LAB strains (KM4, KM14, KMP17, and KM35) exhibited significant inhibitory effects against *S. aureus* ATCC9144, *S. aureus* FBS200, and *Listeria monocytogenes* CICC 21633, as illustrated in Figure 2. The inhibition of *S. aureus* ATCC9144 (Figure 2A): All four strains demonstrated robust inhibitory activity against *S. aureus* ATCC9144, with minimal changes in efficacy after 12 h. Strain *L. sakei* KMP17 showed the strongest activity, producing inhibition zones of 13.17 ± 0.35 mm at 12 h and 14.79 ± 0.43 mm at 48 h. Strains KM4, KM14, and KM35 achieved inhibition zones of 11.35 ± 0.65 mm, 12.37 ± 0.73 mm, and 13.92 ± 0.56 mm, respectively, at 48 h.

Inhibition of *S. aureus* FBS200 (Figure 2B): The CFS of all four strains displayed time-dependent antimicrobial activity against *S. aureus* FBS200. While inhibition zones at 12 h were modest, efficacy varied with fermentation time. *L. sakei* KMP17 exhibited the strongest inhibition overall, with a zone of 17.93 ± 0.65 mm at 48 h. KM4 reached peak activity at 24 h (14.74 ± 1.08 mm), KM14 showed weaker activity (13.73 ± 0.41 mm at 48 h), and KM35 achieved optimal inhibition at 36 h (15.37 ± 1.26 mm).

Inhibition of *Listeria monocytogenes* CICC 21,633 (Figure 2C): Antagonistic activity against *L. monocytogenes* improved with prolonged cultivation, peaking at 36 h. Strain KM35 initially showed a smaller inhibition zone (16.48 ± 0.94 mm at 12 h) but demonstrated superior efficacy at later time points, reaching 28.26 ± 0.18 mm by 48 h. Strains KM4, KM14, and *L. sakei* KMP17 achieved maximal inhibition at 36 h, with zones of 24.47 ± 1.06 mm, 21.26 ± 0.67 mm, and 22.88 ± 1.10 mm, respectively.

### 3.5. Screening of High-Efficiency PAT-Degrading LAB

The PAT content was quantified via HPLC using the standard curve established in Figure S1, with results summarized in Figure S4. All four strains demonstrated robust survival under high PAT concentrations (40 μg/mL), indicating strong PAT tolerance. Furthermore, all strains exhibited significant PAT degradation capabilities. In the control group (uninoculated MRS medium), the PAT concentration remained at 38.77 μg/mL after 24 h. Strain KM4 displayed the highest degradation efficiency, reducing PAT to 24.50 μg/mL (degradation rate: 36.8%). Strains KM14, *L. sakei* KMP17, and KM35 reduced PAT to 25.87 μg/mL (degradation rate: 33.27%), 30.75 μg/mL (20.67%), and 26.24 μg/mL (32.32%), respectively.

### 3.6. Degradation of PAT by L. sakei KMP17

**Figure 3:**
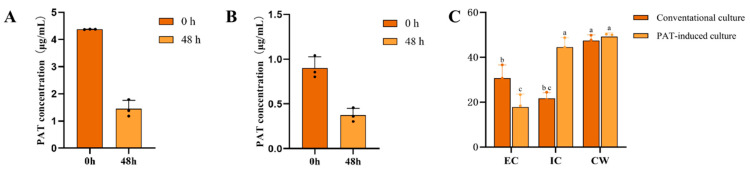
PAT removal by *L. sakei* KMP17. Viability-Dependent Degradation (**A**); Heat-Inactivated Degradation (**B**); Cellular Components (**C**). Different letters on the error bars indicate statistically significant differences (*p* < 0.05). EC, extracellular enzymes; IC, intracellular enzymes; CW; the cell wall. The scatter points overlaid on the bars denote the values of individual biological replicates within each group.

To evaluate the PAT degradation capability of viable *L. sakei* KMP17 cells, experiments were conducted in an MRS reaction system containing 5 μg/mL PAT. As shown in Figure 3A, the initial PAT concentration (0 h) was 4.37 μg/mL. After 48 h of incubation, the PAT content in the medium decreased to 1.45 ± 0.31 μg/mL, with a degradation rate of 66.82 ± 7.11% attributed to viable cells.

For the PAT removal capacity of heat-inactivated *L. sakei* KMP17 Cells. The PAT adsorption capacity of heat-inactivated *L. sakei* KMP17 cells was investigated in a PBS reaction system containing 1 μg/mL PAT, as shown in Figure 3B. The initial PAT concentration (0 h) was 0.90 ± 0.13 μg/mL, decreasing to 0.37 ± 0.08 μg/mL after 48 h of incubation. This corresponds to a PAT removal rate of 58.39 ± 8.59% by heat-inactivated cells. Despite the relatively high removal rate, the absolute PAT reduction was only 0.53 μg/mL, significantly lower than the degradation capacity observed in viable cells (refer to Section 3.2). These results indicate that heat-inactivated *L. sakei* KMP17 cells can adsorb trace amounts of PAT, but metabolic activity remains critical for efficient PAT elimination.

As shown in Figure 3C, extracellular enzymes (EC), intracellular enzymes (IC), and the cell wall (CW) of *L. sakei* KMP17 all participated in the PAT removal process. PAT-induced cultivation may stimulate the production of enzymes specifically involved in PAT degradation. However, the absolute PAT removal levels by individual components were relatively low. After 24 h of co-cultivation, the intracellular fraction from the induced group exhibited the highest PAT removal capacity (1.02 μg/mL), while the cell wall demonstrated the highest removal efficiency among all components.

### 3.7. Optimization of Fermentation Conditions for L. sakei KMP17

**Figure 4:**
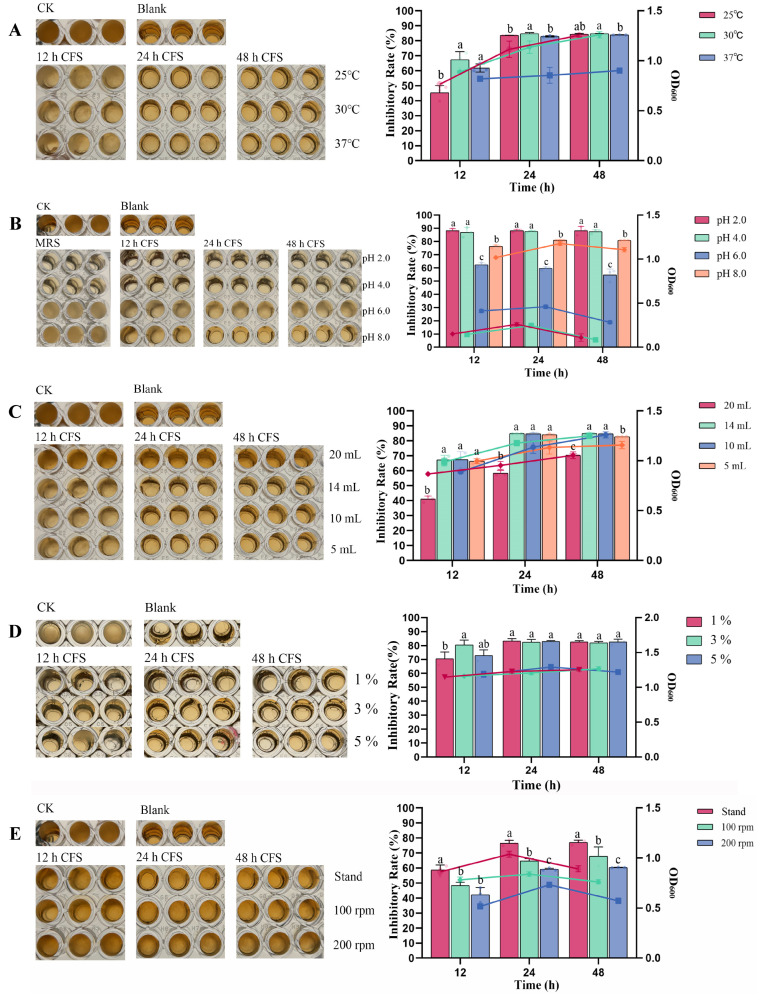
Effect of temperature (**A**), initial pH (**B**), culture volume (**C**), inoculum volume (**D**), agitation speed (**E**) on the growth and antifungal activity of KMP17 against PAT producing fungi *P. expansum*. Microplate assay demonstrating the antifungal activity of KMP17 against *P. expansum*, OD_600_ values and inhibition rate of KMP17 under different conditions. The bar graph shows the inhibition rate of KMP17 against PAT-producing fungi under different conditions, while the line plot depicts the corresponding OD_600_ values. The scatter points overlaid on the bars represent each individual data point within the groups. Different letters on the error bars indicate statistically significant differences within sampling time (*p* < 0.05).

The effects of inoculum size on the growth and antifungal activity of strain KMP17 are shown in Figure 4A. Regarding growth characteristics, the OD_600_ values of all inoculum size groups (1%, 3%, and 5%) exceeded 1.0 between 12 h and 48 h of cultivation, indicating that the strain reached the stationary phase. For antibacterial activity, at 12 h, the CFS from the 3% inoculum group exhibited the highest inhibitory rate (80.54 ± 3.39%), followed by the 1% (70.44 ± 4.98%) and 5% (72.80 ± 4.16%) groups. By 24 h, no significant differences in inhibitory rates were observed among groups, though all maintained strong antibacterial activity, with rates exceeding 82%.

The effect of temperature on the growth and antifungal activity of strain KMP17 is shown in Figure 4B. Significant turbidity changes were observed in all three biological replicates of the positive control group (CK), while hyphal formation accompanied by slight turbidity was detected in the 12 h fermentation broth cultured under three temperature gradients. The inhibition rate of the 25 °C treatment group (45.27 ± 4.89%) was significantly lower than those of other temperature groups (*p* < 0.05), with its corresponding OD_600_ value also remaining at a relatively low level, suggesting that the metabolic activity of the strain was restricted at 25 °C, resulting in limited synthesis of antimicrobial substances within 12 h. After 24 h of cultivation, the inhibition rates of CFS under different temperatures increased substantially, with the 30 °C group achieving an inhibition rate of approximately 84%. Concurrently, growth curve analysis revealed that the OD_600_ values of the 25 °C and 30 °C groups exhibited continuous increases during the 12–48 h cultivation period, whereas the 37 °C group entered the stationary growth phase as early as 12 h.

The effect of liquid volume on the growth and antimicrobial activity of strain KMP17 is shown in Figure 4C. As illustrated in Panel a, hyphal formation accompanied by slight turbidity was observed in the detection wells containing sterile fermentation broth from the 20 mL liquid volume group. The inhibition rate of this group was significantly lower than those of other groups (*p* < 0.05), measuring 41.02 ± 2.13% at 12 h, but increased markedly to 70.34 ± 1.05% by 48 h. Concurrently, the OD_600_ values of the 20 mL group remained lower compared to other groups, likely due to higher oxygen levels under this condition, which suppressed the growth rate of the strain and impaired the metabolic synthesis of antimicrobial substances. During the 12–24 h cultivation period, the antimicrobial activity of all four groups exhibited an upward trend, with inhibition rates increasing from approximately 65% to over 80% in the 14 mL, 10 mL, and 5 mL liquid volume groups. Notably, the 14 mL group demonstrated the highest inhibition rate (84.85 ± 0.04%). By 48 h, all groups maintained high inhibition rates. However, the 5 mL group showed a slight decline in inhibition rate, decreasing from 84.02 ± 0.64% to 82.82 ± 0.10%.

The impact of agitation speed on the growth and antimicrobial activity of strain *L. sakei* KMP17 is illustrated in Figure 4D. As shown in Panel a, hyphal formation and turbidity were observed in detection wells containing 12 h sterile fermentation broth. In contrast, only the 200 rpm experimental group exhibited noticeable hyphae in wells treated with 24 h and 48 h sterile fermentation broth. Regarding growth kinetics, OD_600_ values indicated that the strain entered the stationary growth phase by 12 h, with a subsequent rise and decline in biomass between 12–48 h. The static culture group demonstrated optimal growth, achieving the highest OD_600_ values, whereas suboptimal growth under 200 rpm conditions suggested a potential preference for anaerobic environments.

Antifungal activity correlated with growth performance. At 12 h, the inhibition rate of CFS from the static culture group (58.61 ± 3.47%) was significantly higher than those of the 100 rpm (48.29 ± 2.29%) and 200 rpm (42.24 ± 4.91%) groups. During the 12–24 h cultivation period, antimicrobial activity increased across all groups, with the static culture group reaching 76.61 ± 1.85% inhibition at 24 h, significantly surpassing other groups. By 48 h, significant differences (*p* < 0.05) in inhibition rates were observed: the static group maintained the highest rate (77.03 ± 1.51%), followed by the 100 rpm (67.97 ± 6.13%) and 200 rpm (60.36 ± 0.16%) groups.

The impact of initial medium pH on the growth and antifungal activity of strain *L. sakei* KMP17 is shown in Figure 4E. Similarly to strains KM4 and KM14, the optimal fermentation pH for *L. sakei* KMP17 ranged between 6.0 and 8.0. Under acidic conditions (pH 2.0–4.0), all experimental groups exhibited inhibition rates exceeding 85%, indicating strong suppression of fungal and bacterial growth in such environments. At pH 6.0, despite inhibited growth of *L. sakei* KMP17, both OD_600_ values and inhibition rates were higher than those of KM4 and KM14, aligning with previous findings on *L. sakei* KMP17’s superior acid tolerance. Specifically, the inhibition rate of 12 h CFS was 62.35 ± 1.58%, decreasing slightly to 59.64 ± 1.06% at 24 h and 54.62 ± 4.50% at 48 h. At pH 8.0, *L. sakei* KMP17 achieved optimal growth (OD_600_ > 1.0) and sustained high antifungal efficacy: inhibition rates ranged from 76.17 ± 1.10% to 81.12 ± 0.15% during 12–48 h. Detection wells supplemented with CFS remained clear, suggesting that neutral-to-alkaline conditions favor robust bacterial growth and stable synthesis of antifungal metabolites.

### 3.8. Whole Genome Analysis of L. sakei KMP17

**Figure 5:**
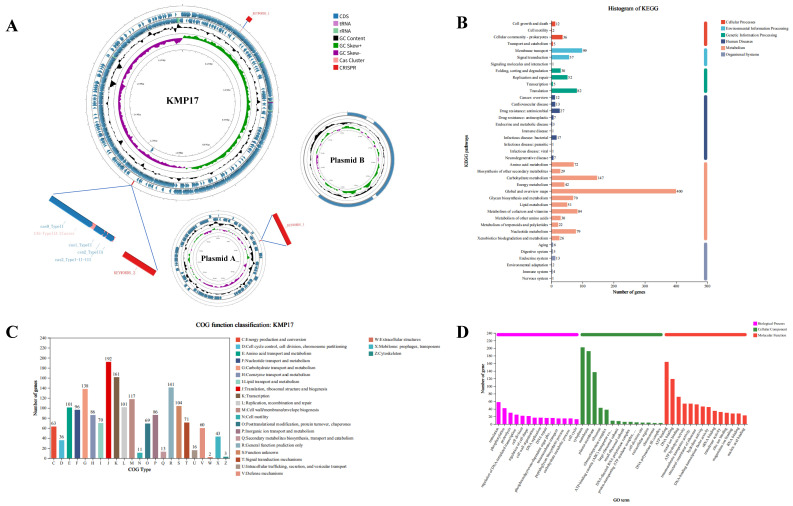
Genomic circular map and functional annotations of *L. sakei* KMP17 (**A**). The outermost circle of the circular plot indicates genome size; the second circle represents CRISPR/Cas related genes, the third and fourth circulars represent CDS on the positive and negative strands, respectively; fifth circle represents GC content; and the innermost circle shows a GC skew ([G − C]/[G + C]) plot of the genome. KEGG orthology categories (**B**), COG functional classifications (**C**), and GO annotation results (**D**) of *L. sakei* KMP17.

*L. sakei* KMP17 harbors a single circular chromosome (1,959,226) with 41.1% G + C content, and two circular plasmids, plasmid 1 (55,356 bp) with G + C content of 39.1%, and plasmid 2 (2271 bp) with G + C content of 36.5%, as illustrated in Figure 5A. The complete genome spans 2,016,753 bp with an average G + C content of 41.08%. It encompasses a total of 1970 genes accounting for approximately 86.97% of the genome length. Noncoding RNA prediction using RNAmmer1.2 identified various types including tRNA (65 in number), rRNA (21 in number comprising seven copies each of the 5S rRNA, 16S rRNA and 23 S rRNA), as well as sRNA (10 in number) species.

There were 1471 genes (74.7% of the total) annotated to KEGG pathways in *L. sakei* KMP17, which were distributed across six categories and 39 functional groups (Figure 5B). Specifically, there are 1052 genes that are involved in metabolism, 147 genes that are involved in carbohydrate metabolism, and 84 genes that are involved in cofactors and vitamins. The processes of sugar uptake, catabolism, and glycogen synthesis indicate a robust carbon metabolism, which ensures a sustained energy supply. Additionally, the enzymatic reactions and physiological stability of the organism may contribute to probiotic functions through vitamin synthesis.

A total of 1618 (82.13%) of the 1970 protein-coding genes of *L. sakei* KMP17 were annotated into 23 COG subcategories (Figure 5C). Four primary classes were designated for these categories: 454 genes were involved in information processing; 385 genes were involved in cellular structure and dynamics; 653 genes were involved in metabolism; and 288 genes were poorly characterized or did not have a known function. According to protein function, 1104 genes (56.05% of the total) in *L. sakei* KMP17 were functionally categorized using the GO database (Figure 5D). 905 genes were engaged in molecular function, 610 in cellular components, and 607 in biological processes. The top three categories included cytoplasm (202 genes), cell membrane (192 genes), and ATP-binding (164 genes). GO annotations help interpret the biological roles encoded by genes.

#### 3.8.1. Analysis of Secondary Metabolite Biosynthesis

**Figure 6:**
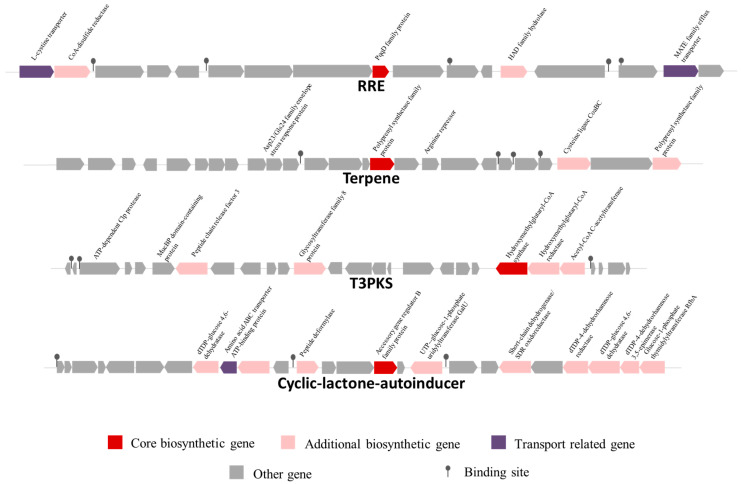
Linear maps of secondary metabolite synthesis gene clusters in *L. sakei* KMP17. Linear map of the RRE-containing, terpene, T3PKS, and cyclic-lactone-autoinducer synthesis gene cluster.

AntiSMASH analysis predicted two potential secondary metabolite BGCs in *L. sakei* KMP17: RRE-containing, terpene, T3PKS, cyclic lactone autoinducer (Figure 6). The RRE-containing BGC spans nucleotides 348,769 to 369,123 (total length: 20,354 nt), encompassing 18 genes. No homologous clusters were identified in the database. The T3PKS BGC including cyclic lactone autoinducer spans nucleotides 1,507,031 to 1,553,375 (total length: 46,344 nt), containing 46 genes with 23% similarity to polysaccharide-related clusters.

#### 3.8.2. Analysis of Antimicrobial Resistance and Virulence Genes

Bacteria develop resistance to antibiotics through mutations or the acquisition of resistance genes, which has increasingly compromised the treatment of infections. To identify and characterize antimicrobial resistance (AMR) genes, we annotated the genome of *L. sakei* KMP17 using the Comprehensive Antibiotic Resistance Database (CARD). CARD integrates antibiotic resistance-associated reference genes from diverse organisms, genomes, and plasmids, serving as a critical resource for studying resistomes and resistance mechanisms in environmental, human, and animal microbiomes [31]. With a threshold of identity ≥80%, CARD annotation revealed four AMR genes in the KMP17 genome (Table S3). The absence of plasmid-mediated resistance genes, despite their detection in the genome and phenotypic resistance profiles, suggests that these AMR traits are intrinsic rather than acquired through horizontal gene transfer. In contrast, the presence of resistance plasmids would necessitate vigilance regarding potential horizontal dissemination [32]. Notably, no plasmid-borne resistance genes were identified in *L. sakei* KMP17. Phenotypic assays demonstrated resistance to six antibiotics: nalidixic acid (NA), amikacin (AMK), kanamycin (KAN), vancomycin (VAN), streptomycin (STR), and gentamicin (GEM). Studies indicate that intrinsic resistance (e.g., to vancomycin) is common among lactic acid bacteria (LAB) and represents an inherent trait that enables their safe application in combination therapies [33]. The majority of AMR traits observed in *L. sakei* KMP17 align with intrinsic LAB resistance profiles, implying a relatively low risk of AMR gene transfer under the tested conditions.

The ResFinder database catalogs experimentally validated antimicrobial resistance (AR) genes and enables the identification of resistance determinants from bacterial DNA sequences using ResFinder 4.0. Comparison of *L. sakei* KMP17’s genome against this database revealed no chromosomally encoded AR genes; however, a putative resistance gene, *clpL*, was predicted on plasmid A. This gene exhibited 98.16% sequence identity and 100% coverage with the *clpL* gene carried by plasmid pLM58 in Listeria monocytogenes AT3E.

Virulence factors, defined as microbial components that enhance pathogenicity or promote infection in specific hosts, include bacterial toxins, cell surface adhesins, protective proteins, surface carbohydrates, and hydrolytic enzymes. The Virulence Factors Database (VFDB) provides a comprehensive repository for pathogen-associated virulence data. Using a sequence identity threshold of ≥70%, genomic alignment of *L. sakei* KMP17 against VFDB identified two virulence genes: *clpP* and hyaluronic acid capsule (Table S4). The *clpP* gene, encoding a serine protease involved in proteolysis, is essential for bacterial growth under stress conditions [34]. Hemolysins, common virulence factors in pathogenic bacteria, disrupt erythrocytes and contribute to conditions such as edema and anemia. Notably, no hemolysin-associated virulence factors were detected in *L. sakei* KMP17, consistent with its previously reported hemolytic inactivity. These bioinformatics-based findings validate the safety profile of *L. sakei* KMP17, supporting its potential application in probiotic research.

#### 3.8.3. Genetic Composition of Genes Related to Probiotic Characteristics

The tolerance of probiotics to acid and bile salts is a critical factor in their survival during digestion and colonization in the gastrointestinal tract. Several genes associated with encoding cyclopropane synthase (gene0044), CTP synthase (gene1723), cholylglycine hydrolase (gene0211), and ornithine carbamoyltransferase (gene0405) were found in its genome, and eight genes associated with encoding proton translocating ATPase (F1F0-ATPase) were identified in the *L. sakei* KMP17 genome. Some other genes related to acids and bile salts were also found, which allowed the strain to demonstrate tolerance to acid and bile salts. Bacterial adhesion is one mechanism that influences probiotics’ capacity to colonize the gut. The *L. sakei* KMP17 genome was analyzed and found to have genes coding for adhesion-related proteins. There was one gene encoding a cell wall anchoring protein, two genes related to trimeric autotransporter adhesin, and five genes encoding elongation factors (Table 1).

*L. sakei* KMP17 contains a substantial number of genes that encode compounds with immunomodulatory and antioxidant properties (Table 2). Nine genes encoded the SDR family, which was the most numerous. Next, genes that encode thioredoxin, peptide-methionine (R)-S-oxidoreductase, and glutaredoxin were identified. The SDR family of NAD(P)-dependent oxidoreductases has numerous pathways that are crucial for the host’s antioxidant defense, such as direct ROS scavenging, antioxidant molecule regeneration, and antioxidant gene expression control. In conclusion, the presence of genes *dltA*, *dltB*, *dltC*, and *dltD*, which are involved in the synthesis of D-alanine, as well as *tagF*, *tagT*, *yfhO*, and *licD*, which are involved in teichoic acid biosynthesis, indicates that *L. sakei* KMP17 has immunomodulatory capabilities. These genes may serve to regulate the immune response of the host. The set of genes that are associated with bile salt tolerance, pH survival, cold and heat stress, and osmotic stress, was examined and identified in *L. sakei* KMP17. This discovery suggests that these genes are crucial for the survival and adaptation of the organism to the gastrointestinal environment.

## 4. Discussion

This study focused on four LAB strains previously isolated from pickles for their antifungal activity, with *L. sakei* KMP17 selected for further investigation due to its superior probiotic potential. Strain identification revealed two *W. cibaria* (KM4, KM14), one *L. sakei* KMP17, and one *Leuconostoc mesenteroides* KM35. All strains exhibited moderate survival under low pH (3.0) and 0.3% oxgall, though their viability was lower than reported for other LAB, such as *Enterococcus durans* L61, which survives poorly in gastric conditions but thrives in simulated intestinal fluid [35]. Hydrophobicity (≥20% for chloroform, ≥30% for xylene) and auto-aggregation (≥45% after 24 h) suggested strong adhesion potential, a critical trait for intestinal colonization [36,37].

Antibiotic resistance is now recognized as a critical issue in modern medicine. Ac-cording to the European Food Safety Authority (EFSA), the horizontal transfer risk of intrinsic antibiotic resistance in bacteria is considered low, whereas acquired resistance is classified as high-risk. Safety assessments indicated susceptibility to ampicillin/sulbactam, tetracycline, and erythromycin, but resistance to nalidixic acid and vancomycin. In assessing the antibiotic resistance safety of strain *L. sakei* KMP17, we focused our discussion on its resistance to nalidixic acid and vancomycin. These two forms of resistance are widely regarded as intrinsic, stemming, respectively, from the cell wall characteristics of Gram-positive bacteria and their inherent cell wall precursor structure. The study also observed tolerance of the strain to several aminoglycoside antibiotics (amikacin, kanamycin, streptomycin, and gentamicin). The underlying mechanism—whether intrinsic or acquired—requires clarification through subsequent molecular genetic analysis. The current safety inference is primarily based on the evaluation of well-defined intrinsic resistance.

Hemolytic activity is a critical safety indicator for LAB. While *L. sakei* KMP17 showed no hemolytic activity, KM4 and KM14 exhibited α-hemolysis and KM35 displayed β-hemolysis, necessitating further safety evaluation. All strains demonstrated significant antagonism against pathogens (*S. aureus* ATCC9144, FBS200; *Listeria monocytogenes* CICC21633), highlighting their probiotic potential, with *L. sakei* KMP17 outperforming others in comprehensive traits. The four LAB strains also degraded PAT in MRS broth (40 μg/mL, 24 h), with degradation rates of 36.8% (KM4), 33.27% (KM14), 20.67% (*L. sakei* KMP17), and 32.32% (KM35). Based on prior findings, *L. sakei* KMP17 exhibited the most prominent probiotic properties and was confirmed to lack hemolytic activity through safety assessments. Although its PAT degradation efficiency (9.25 μg/mL reduction over 24 h) was comparatively lower than other strains, it demonstrated preliminary potential for PAT degradation. Considering its balanced profile of probiotic characteristics, safety, and biodegradation performance, *L. sakei* KMP17 was selected as the target strain for further mechanistic studies.

Investigating the mechanism of PAT degradation by *L. sakei* KMP17 provides theoretical guidance for its practical application. Studies have revealed two primary pathways for microbial biodegradation of PAT: enzymatic degradation mediated by metabolites synthesized by bacterial strains, and adsorption through peptidoglycan or polysaccharides in microbial cell walls [38].

Mechanistic studies on *L. sakei* KMP17 revealed that viable cells drove PAT removal (66.82 ± 7.11% in 5 μg/mL PAT), significantly surpassing heat-killed cells, cell walls, or extracellular/intracellular fractions. PAT induction enhanced degradation efficiency, aligning with reports that microbial viability is critical for PAT detoxification, as seen in *Saccharomyces cerevisiae* and *L. casei* YZU01 [23,39]. Three potential mechanisms were considered: cell wall adsorption, intracellular enzymatic degradation, and extracellular enzyme-mediated breakdown. Although heat-killed *S. cerevisiae* showed comparable PAT adsorption to viable cells [40,41], the limited PAT removal by *L. sakei* KMP17’s heat-killed biomass or cell walls (thick peptidoglycan layer) suggested adsorption was secondary to metabolic activity. Notably, intracellular and extracellular fractions of PAT-induced *L. sakei* KMP17 exhibited enhanced degradation, implying inducible enzymatic pathways. Enzyme-based detoxification, being more efficient and consumer-acceptable, underscores *L. sakei* KMP17’s potential for PAT bioremediation. Standardization of Fermentation Conditions revealed *L. sakei* KMP17’s robustness across inoculum sizes, temperatures, agitation speeds, and oxygen levels (modified by medium volume), with optimal growth and antifungal activity at pH 6–8 under microaerobic conditions.

Genomic analysis of *L. sakei* KMP17 identified a circular chromosome (2,016,753 bp, GC 41.08%) and two plasmids (A: 55,356 bp; B: 2271 bp), with 1970 annotated genes. CAZy annotation detected 49 carbohydrate-active enzymes (predominantly glycoside hydrolases), supporting metabolic versatility. Despite four putative antimicrobial resistance (AMR) genes (CARD), none were plasmid-associated, suggesting low horizontal transfer risk. VFDB analysis identified two virulence genes (*clpP*, hyaluronic acid capsule), but no hemolysins, consistent with its safety profile. The results indicate that *L. sakei* KMP17 has the potential to serve as a probiotic, with the potential to contribute to gastrointestinal health and pathogen protection, as well as to be characterized as an antioxidant and immunomodulator. A variety of probiotic marker genes were identified in *L. sakei* KMP17 (as listed in Table 1 and Table 2). The genomic levels of its probiotic functions were suggested by this discovery. This further supports the idea that *L. sakei* KMP17 may be a prospective candidate probiotic for application in the food industry.

## 5. Perspectives

Probiotics, defined as microorganisms conferring health benefits to humans and animals, hold significant potential in food, healthcare, and agriculture. LAB, a prominent probiotic group with diverse physiological functions, are widely utilized. While the four LAB strains in this study (*W. cibaria* KM4, KM14; *L. sakei* KMP17; *Leuconostoc mesenteroides* KM35) demonstrated promising in vitro probiotic traits, their efficacy and safety require rigorous validation through in vivo studies.

All strains exhibited potent antifungal and antibacterial activity, attributed to secreted metabolites rather than direct microbial antagonism, as cell-free supernatants were used in inhibition assays [42]. Further investigations are warranted to elucidate the specific antimicrobial compounds and interspecies antagonistic interactions.

Notably, all four strains degraded PAT, though this study only preliminarily explored the mechanism in *L. sakei* KMP17. Degradation pathways may vary across species, necessitating comparative analyses of PAT metabolism and detoxification products in KM4, KM14, and KM35. While *L. sakei* KMP17 primarily relied on viable cell metabolism for PAT removal, the genetic basis of PAT degradation remains poorly understood. Future work could integrate genomic data to identify key genes or enzymatic pathways involved.

Under PAT stress, LAB may induce stress-responsive enzymes with specific detoxification capabilities, offering potential for food safety applications. However, critical questions persist regarding PAT-induced gene regulation, enzyme structure–function relationships, and catalytic mechanisms. Multidisciplinary approaches, including transcriptomics, proteomics, and molecular biology, are essential to unravel these mechanisms, advancing the development of enzymatic detoxification strategies that are both efficient and consumer-acceptable.

## Figures and Tables

**Table 1:** Predicted genes involved in the potential probiotic properties of *L. sakei* KMP17.

Gene	Gene ID	Gene Description
Heat stress		
*hslU*	gene1066	ATP-dependent protease ATPase subunit HslU
*hslV*	gene1067	ATP-dependent protease subunit HslV
*hslO*	gene1690	Hsp33 family molecular chaperone HslO
*dnaJ*	gene1298	Molecular chaperone DnaJ
*dnaK*	gene1299	Molecular chaperone DnaK
*GRPE*	gene1300	Molecular chaperone GrpE (heat shock protein HSP-70)
*hrcA*	gene1301	Heat-inducible transcriptional repressor HrcA
*HSP20*	gene0049	Hsp20/alpha crystallin family protein
*clpA*	gene0208	ATP-dependent Clp protease ATP-binding subunit
*clpA*	pA_gene0060	ATP-dependent Clp protease ATP-binding subunit
*clpB*	gene1115	ATP-dependent chaperone ClpB
*clpA*	gene1539	ATP-dependent Clp protease ATP-binding subunit
*clpA*	gene1855	ATP-dependent Clp protease ATP-binding subunit
*clpP*	gene0599	ATP-dependent Clp endopeptidase proteolytic subunit ClpP
*clpX*	gene1138	ATP-dependent Clp protease ATP-binding subunit ClpX
*htpX*	gene1713	Zinc metalloprotease HtpX
*groES*	gene0391	Co-chaperone GroES
*groEL*	gene0392	Chaperonin GroEL
*lysS*	gene1696	RNA-binding S4 domain-containing protein
Cold stress		
*cspA*	gene0845	Cold-shock protein
*cspA*	gene1028	Cold shock domain-containing protein
*cspA*	gene1231	MULTISPECIES: cold-shock protein
*cspA*	gene1642	MULTISPECIES: cold-shock protein
Ionic and heavy metal stress		
*cutC*	gene1514	Copper homeostasis protein CutC
*corA*	gene0036	Mg^2+^ and Co^2+^ transporter CorA
*corA*	gene0547	Mg^2+^ and Co^2+^ transporter CorA
*corA*	pA_gene0006	Mg^2+^ and Co^2+^ transporter CorA
*zurR*	gene0098	Fe^2+^ or Zn^2+^ uptake regulation protein Fur/Zur
*perR*	gene0530	Fe^2+^ or Zn^2+^ uptake regulation protein Fur/Zur
*furR*	gene1104	Fe^2+^ or Zn^2+^ uptake regulation protein Fur/Zur
pH stress		
*Asp23*	gene0160	Asp23/Gls24 family envelope stress response protein
*Asp23*	gene0161	Asp23/Gls24 family envelope stress response protein
*Asp23*	gene0748	Asp23/Gls24 family envelope stress response protein
*Asp23*	gene0771	Asp23/Gls24 family envelope stress response protein
*clcA*	gene0385	H^+^/Cl^-^ antiporter ClcA
*clcA*	gene1719	H^+^/Cl^-^ antiporter ClcA
*clcA*	gene1720	H^+^/Cl^-^ antiporter ClcA
*nhaC*	gene1912	Na^+^/H^+^ antiporter NhaC
*nhaD*	gene0333	Na^+^/H^+^ antiporter NhaD or related arsenite permease
*nhaP*	gene0267	NhaP-type Na^+^/H^+^ or K^+^/H^+^ antiporter
*nhaP*	gene0269	NhaP-type Na^+^/H^+^ or K^+^/H^+^ antiporter
*nhaP*	gene0268	NhaP-type Na^+^/H^+^ or K^+^/H^+^ antiporter
*nhaP*	gene1410	NhaP-type Na^+^/H^+^ or K^+^/H^+^ antiporter
*atpC*	gene1203	FoF1-type ATP synthase, epsilon subunit
*atpD*	gene1204	FoF1-type ATP synthase, beta subunit
*atpG*	gene1205	FoF1-type ATP synthase, gamma subunit
*atpA*	gene1206	FoF1-type ATP synthase, alpha subunit
*atpH*	gene1207	FoF1-type ATP synthase, delta subunit
*atpF*	gene1208	FoF1-type ATP synthase, membrane subunit B
*atpE*	gene1209	FoF1-type ATP synthase, membrane subunit C
*atpB*	gene1210	FoF1-type ATP synthase, membrane subunit A
Osmotic stress		
*opuA*	gene0691	Osmoprotectant transport system ATP-binding protein
*opuA*	gene1956	Osmoprotectant transport system ATP-binding protein
*opuC*	gene0693	Osmoprotectant transport system substrate-binding protein
*opuC*	gene1955	Osmoprotectant transport system substrate-binding protein
*opuBD*	gene0692	Osmoprotectant transport system permease protein
*opuBD*	gene0694	Osmoprotectant transport system permease protein
*TC.APA*	gene0119	Basic amino acid/polyamine antiporter, APA family
*TC.APA*	gene0188	Basic amino acid/polyamine antiporter, APA family
*TC.APA*	gene0393	Basic amino acid/polyamine antiporter, APA family
*TC.APA*	gene1296	Basic amino acid/polyamine antiporter, APA family
*TC.APA*	gene1497	Basic amino acid/polyamine antiporter, APA family
Bile salt stress		
*cfa*	gene0044	Cyclopropane-fatty-acyl-phospholipid synthase
*yxeI*	gene0211	Choloylglycine hydrolase family protein
*pgk*	gene0682	Phosphoglycerate kinase
*argF*	gene0405	Ornithine carbamoyltransferase
*pyrG*	gene1723	CTP synthase
*argS*	gene1493	Arginine-tRNA ligase
Adhesion		
*sadA*	gene1924	LPXTG cell wall anchor domain-containing protein
*sadA*	gene0166	Trimeric autotransporter adhesin
*sadA*	gene0228	Trimeric autotransporter adhesin
*eno*	gene0684	Phosphopyruvate hydratase
*tuf*	gene1140	Elongation factor Tu
*efp*	gene0287	Elongation factor P
*efp*	gene0747	Elongation factor P
*tsf*	gene1340	Elongation factor Ts
*fusA*	gene1845	Elongation factor G

**Table 2:** Putative genes of antioxidant and immunomodulation enzyme in *L. sakei* KMP17.

Gene	Gene ID	Gene Description
Antitumor or antioxidant		
*arcA*	gene0404	Arginine deiminase
*argR*	gene0690	Arginine pathway regulatory protein ArgR, repressor of arg regulon
*argR*	gene0755	Arginine repressor
*idnO*	gene0608	SDR family NAD(P)-dependent oxidoreductase
*fabG*	gene1579	SDR family NAD(P)-dependent oxidoreductase
*gdh*	gene0156	SDR family NAD(P)-dependent oxidoreductase
*fabG*	gene0554	SDR family NAD(P)-dependent oxidoreductase
*car*	gene0260	SDR family NAD(P)-dependent oxidoreductase
*ydfG*	gene0606	SDR family NAD(P)-dependent oxidoreductase
*car*	gene1885	SDR family NAD(P)-dependent oxidoreductase
*codA*	gene0348	SDR family oxidoreductase
-	gene1117	SDR family oxidoreductase
*trxA*	gene0245	Thioredoxin
*trxA*	gene0340	Thioredoxin
*trxA*	gene0428	Thioredoxin
*trxA*	pA_gene0047	Thioredoxin
*trxB*	gene0588	Thioredoxin reductase (NADPH) [EC:1.8.1.9]
*trxB*	pA_gene0050	Thioredoxin reductase (NADPH) [EC:1.8.1.9]
*msrB*	gene0941	Peptide-methionine (R)-S-oxide reductase
*msrA*	gene0942	Peptide-methionine (R)-S-oxide reductase
*msrC*	gene0904	L-methionine (R)-S-oxide reductase
*nrdH*	gene1024	Glutaredoxin
Immunomodulation		
*dltA*	gene0430	D-alanine--poly(phosphoribitol) ligase subunit DltA
*dltB*	gene0431	D-alanyl-lipoteichoic acid biosynthesis protein DltB
*dltC*	gene0432	D-alanine--poly(phosphoribitol) ligase subunit DltC
*dltD*	gene0433	D-alanyl-lipoteichoic acid biosynthesis protein DltD
*tagT*	gene0186	Teichoic acid biosynthesis
*tagT*	gene0317	Teichoic acid biosynthesis
*tagT*	gene1595	Teichoic acid biosynthesis
*yfhO*	gene1283	Teichoic acid biosynthesis
*yfhO*	gene1409	Teichoic acid biosynthesis
*yfhO*	gene1667	Teichoic acid biosynthesis
*licD*	gene1590	Teichoic acid biosynthesis
*tagF*	gene1662	Teichoic acid biosynthesis

## Data Availability

The original contributions presented in this study are included in the article/Supplementary Material. Further inquiries can be directed to the corresponding authors.

## Supplementary Materials

The following supporting information can be downloaded at: https://www.mdpi.com/article/10.3390/foods15020234/s1. Table S1: Results of 16SRNA sequence comparison and identification of four LAB strains. Table S2: Antibiotic sensitivity test of the strains. Table S3: Results of CARD annotations of *L. sakei* KMP17. Table S4: Results of VF-related gene in the genomes of *L. sakei* KMP17. Figure S1: Standard curve of patulin concentration by HPLC. Figure S2: Phylogenetic tree based on 16S rRNA gene sequences using the neighbor-joining method showing the position of KMP17 among the related taxa. The bootstrap percentages are based on 1000 replications; only values over 50 % are shown. Bar, 0.5 substitution per 100 nt positions. Figure S3: Hemolytic activity evaluation of four LAB strains was assessed using Columbia agar containing 5% (*w*/*v*) sheep blood for 48h at 30 °C. *L. sakei* KM-17colonies exhibited no hemolysis, whereas *Staphylococcus aureus* ATCC 25923 colonies showed clear lysis of blood cells. *S. aureus* ATCC 25923 (a); is *W. cibaria* KM-4 (b); is *W. cibaria* KM-14 (c); is *L. sakei* KM-17 (d); is *L. mesenteroides* KM-35 (e). Figure S4: Degradation rates of PAT by four LAB strains.
