# Supplementary material for: Evaluation of Probiotic and Antimicrobial Properties of Patulin-Degrading Latilactobacillus sakei KMP17 and Its Fermentation
Source: Foods. 2026 Jan 9;15(2):234. doi: 10.3390/foods15020234 (PMC12840443; doi:10.3390/foods15020234)

Supporting Materials

Evaluation of Probiotic and Antimicrobial Properties of Patulin-Degrading *Latilactobacillus sakei* KMP17 and Its Fermentation

Zi-Qi Yang [1], Xin-Ru Wen [1], Chun-Zhi Jin [2], Taihua Li [1], Feng-Jie Jin [1], Hyung-Gwan Lee [2,*] and Long Jin [1,*]

[1] Co-Innovation Center for Sustainable Forestry in Southern China, College of Ecology and Environment, Nanjing Forestry University, Nanjing 210037, China
[2] Cell Factory Research Centre, Korea Research Institute of Bioscience & Biotechnology (KRIBB), Daejeon 34141, Republic of Korea
* Correspondence: trustin@kribb.re.kr (H.-G.L.); isacckim@alumni.kaist.ac.kr (L.J.)

Table S1. Results of 16SRNA sequence comparison and identification of four LAB strains.

Strains	Closest taxa	Similarity (%)
KM4	*Weissella cibaria* KACC 11862^T	100
KM14	*Weissella cibaria* KACC 11862^T	100
KMP17	*Latilactobacillus sakei* DSM 20017^T	100
KM35	*Leuconostoc mesenteroides* Wikim SH006^T	100

Table S2. Antibiotic sensitivity test of the strains.

Antibiotics	Strain ID			
	KM4	KM14	KMP17	KM35
SAM	S	S	S	S
SH	I	R	I	I
LM	I	I	I	S
NA	R	R	R	R
TCY	S	S	S	S
AMK	I	I	R	I
KAN	R	R	R	I
VAN	R	R	R	R
CHL	I	I	S	I
ERY	S	S	S	S
STR	R	R	R	I
RIF	I	I	S	S
GEM	I	I	R	I
TEC	R	R	I	R

Note. R = Resistant, I = Intermediate, S = Susceptible.

Table S3. Results of CARD annotations of *L. sakei* KMP17.

Gene ID	AMR Gene	Antibiotic Resistance	Resistance Mechanism	Identity (%)
gene1140	*tuf*	Elfamycin antibiotic	Alteration of antibiotic target	74.7
gene1443	*liaR*	Peptide antibiotic	Antibiotic efflux, alteration of antibiotic target	75.6
gene1845	*fusA*	Fusidane antibiotic	Alteration of antibiotic target	72.0
gene1851	*rpoB*	Peptide antibiotic, rifamycin antibiotic	Alteration of antibiotic target, target replacement	72.0

Note. Identity (%): Sequence identity, the percentage of completely identical regions between two sequences. Score: Alignment score, where a higher value indicates higher homology between the two sequences.

Table S4. Results of VF-related gene in the genomes of *L. sakei* KMP17.

Gene ID	Virulence Factor Name	VFDB Accession	Identity (%)
gene0599	ClpP (VF0074)	VFG000077	72.3
gene1576	Hyaluronic acid capsule (VF0244)	VFG000964	70.1

Figure S1. Standard curve of patulin concentration by HPLC.

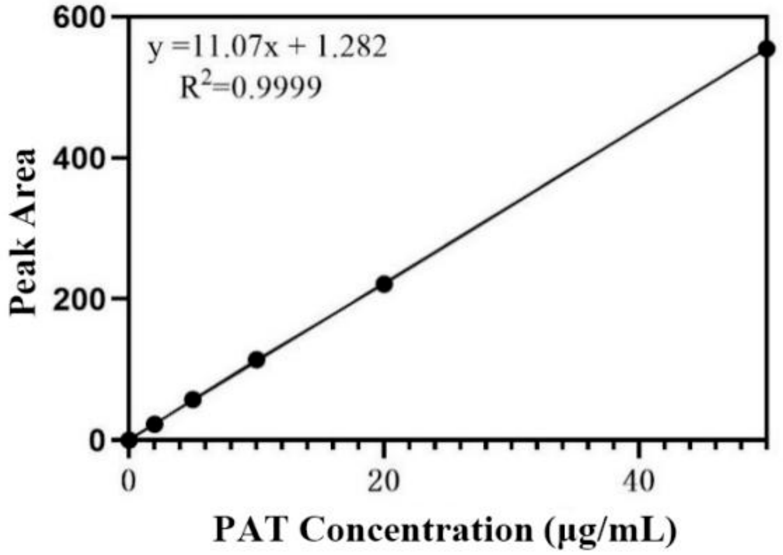

Figure S2. Phylogenetic tree based on 16S rRNA gene sequences using the neighbor-joining method showing the position of KMP17 among the related taxa. The bootstrap percentages are based on 1000 replications; only values over 50 % are shown. Bar, 0.5 substitution per 100 nt positions.

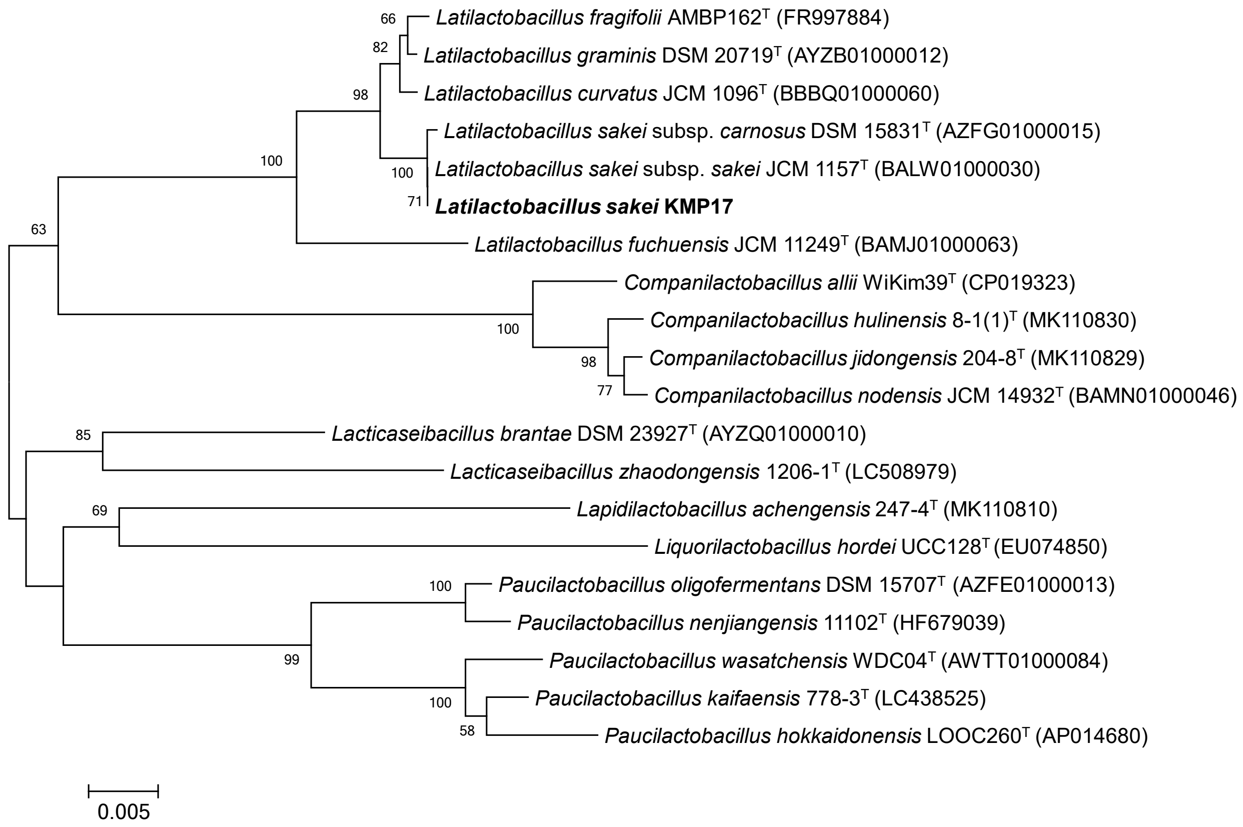

Figure S3. Hemolytic activity evaluation of four LAB strains was assessed using Columbia agar containing 5% (w/v) sheep blood for 48h at 30 °C. *L. sakei* KM-17colonies exhibited no hemolysis, whereas *Staphylococcus aureus* ATCC 25923 colonies showed clear lysis of blood cells. *S. aureus* ATCC 25923 (a); is *W. cibaria* KM-4 (b); is *W. cibaria* KM-14 (c); is *L. sakei* KM-17 (d); is *L. mesenteroides* KM-35 (e).

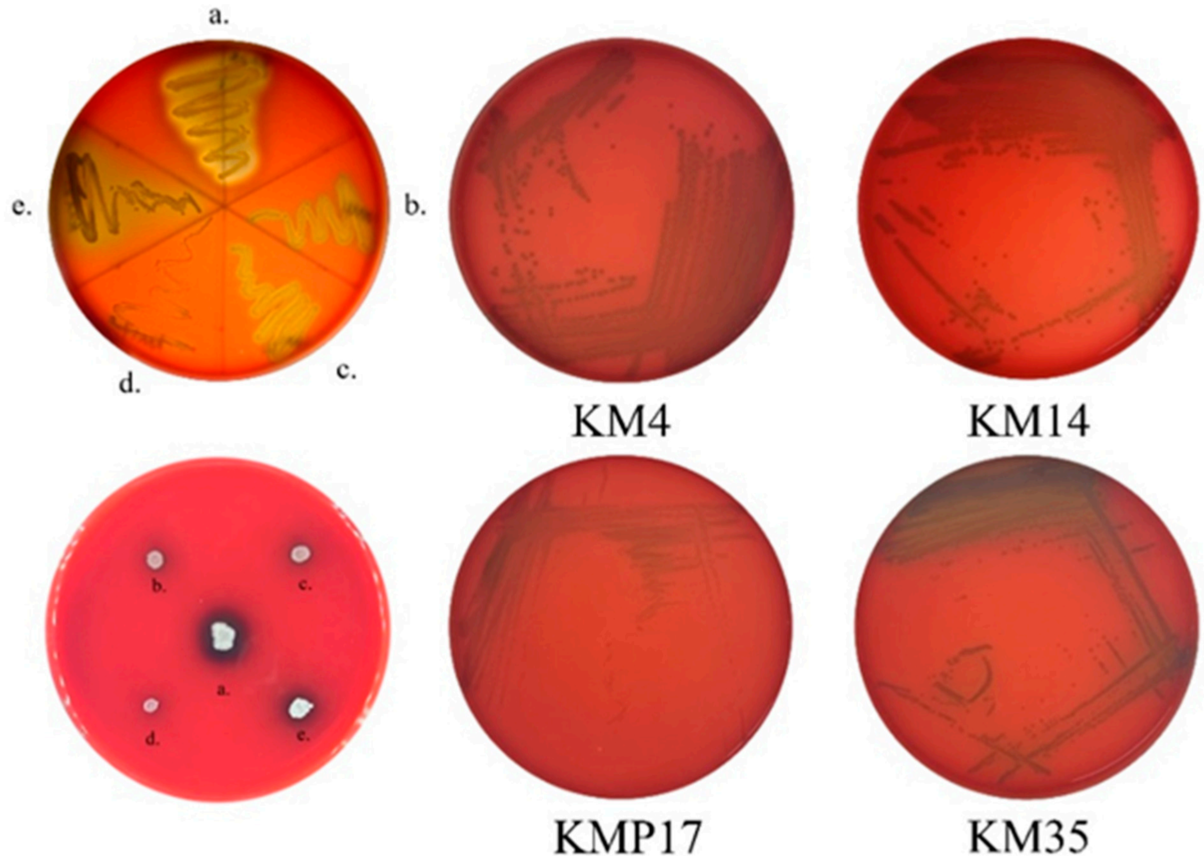

Figure S4. Degradation rates of PAT by four LAB strains.

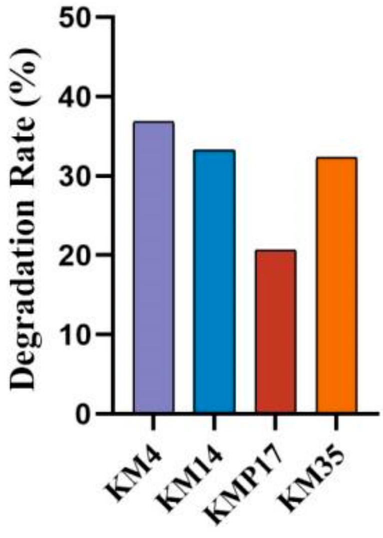

Supplement: Supplementary file 1 [file foods-15-00234-s001.zip › foods-4013634-supplementary.pdf]
